# In Situ Surface Reconstruction and Carbon Encapsulation for High‐Performance Pt‐Lean Catalysts beyond Conventional Core–Shell Designs

**DOI:** 10.1002/smll.202511516

**Published:** 2026-02-11

**Authors:** Jiho Min, Jeong Hee Lee, Keonwoo Ko, Yunjin Kim, Hyelim Park, Mansu Kim, Dongwook Lee, Sung‐Dae Yim, Yun Sik Kang, Joseph T. Hupp, Sung Jong Yoo, Namgee Jung

**Affiliations:** ^1^ Hydrogen Fuel Cell Laboratory Korea Institute of Energy Research (KIER) Daejeon Republic of Korea; ^2^ Center for Hydrogen·Fuel Cell Research Korea Institute of Science and Technology (KIST) Seoul Republic of Korea; ^3^ Graduate School of Energy Science and Technology (GEST) Chungnam National University (CNU) Daejeon Republic of Korea; ^4^ Department of Chemistry Northwestern University Evanston Illinois USA; ^5^ Division of Energy & Environment Technology KIST School University of Science and Technology (UST) Daejeon Republic of Korea

**Keywords:** in situ one‐step process, oxygen reduction reaction, polymer electrolyte membrane fuel cells, porous carbon shell, PtCo alloy, Pt segregation, ultralow Pt

## Abstract

Over recent decades, extensive efforts have aimed to enhance fuel cell performance. Pt alloys with 3d transition metals are particularly attractive for boosting oxygen reduction reaction (ORR) activity via strain and electronic effects. However, their structural instability and high Pt usage hinder practical application. Here, we report a highly active and durable catalyst with reduced Pt cost, achieved by integrating a Pt‐segregated surface and porous carbon shell. Unlike conventional polymer‐coating and carbonization methods, this catalyst is synthesized through a novel ‘in situ one‐step’ process that simultaneously induces Pt segregation and carbon shell formation. This streamlined approach not only simplifies synthesis but also significantly lowers Pt consumption while maintaining superior ORR activity and long‐term durability. As a result, the Pt content is reduced to ∼55% of that in commercial catalysts, while preserving high catalytic activity. Under single‐cell testing, the catalyst exhibits excellent activity and durability, meeting DOE targets even at a Pt loading of 0.02 mg cm^−^^2^, only one‐tenth of conventional loadings (0.2 mg cm^−^^2^). Therefore, this strategy provides a promising pathway toward low‐cost, high‐performance fuel cell catalysts, offering a practical alternative to conventional core–shell or carbon‐coating approaches.

## Graphical abstract

A one‐step in situ fabrication produces Pt‐segregated nanoparticles encapsulated in porous carbon shells. This design drastically reduces Pt content while maintaining high oxygen reduction reaction activity and long‐term durability. The catalyst meets DOE targets even at ultralow Pt loading, presenting a practical pathway toward cost‐effective, high‐performance fuel cells beyond conventional core–shell or carbon‐coating strategies.

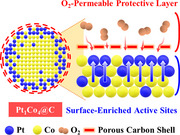

## 1 Introduction

Over the past few decades, extensive research has been conducted on Pt‐based alloy nanoparticle catalysts to enhance fuel cell activity. In particular, alloying Pt with 3d transition metals is well known to significantly improve oxygen reduction reaction (ORR) activity due to strain effects and electronic effects [1, 2, 3, 4, 5, 6, 7, 8, 9, 10]. Among various alloy compositions, Pt_3_M (M = Fe, Co, etc.) has been extensively studied as an optimal composition for high ORR activity, with numerous experimental studies demonstrating its superior performance. However, when applied in practical fuel cells, Pt‐based alloy nanoparticles suffer from structural instability, which leads to performance degradation [7, 8, 9, 10]. During operation, these catalysts are prone to particle aggregation and leaching of 3d transition metals, causing a gradual decline in catalyst activity. To address these issues, researchers have proposed various surface modification strategies aimed at enhancing the durability of alloy nanoparticles while simultaneously reducing the high cost associated with Pt usage.

One of the most widely investigated approaches involves the development of core–shell catalysts, where a 3d transition metal serves as the core and a Pt shell encapsulates the surface [11, 12, 13, 14, 15, 16, 17, 18]. This structure effectively suppresses the dissolution of transition metals while maintaining ORR activity comparable to that of PtM alloy catalysts. However, prolonged electrochemical cycling leads to the gradual dissolution of the Pt shell, exposing the underlying transition metal core to the acidic fuel cell conditions [17, 18]. As a result, the core metal undergoes leaching, ultimately compromising the catalyst's long‐term durability. Moreover, the structural constraint that requires complete encapsulation of the core by the Pt shell limits the achievable Pt decrease, allowing for a maximum decrease of only 38% compared to commercial Pt catalysts. This limitation underscores the challenge of further minimizing Pt consumption in core–shell structures.

To overcome the durability issues associated with core–shell catalysts, an alternative approach has been proposed, involving carbon shell‐coated metal nanoparticles [19, 20, 21, 22]. Carbon shell has emerged as a promising strategy to suppress transition metal dissolution and enhance catalyst durability. However, conventional approaches typically involve carbonization of polymer coatings, which present limitations in precisely controlling the surface structure of the encapsulated catalyst nanoparticles [19, 20, 21, 22, 23]. In particular, this carbon shell has primarily acted as templates that modifies the bulk structure of catalyst particles [23], with limited ability to tailor their surface atomic composition or structure.

**FIGURE 1:**
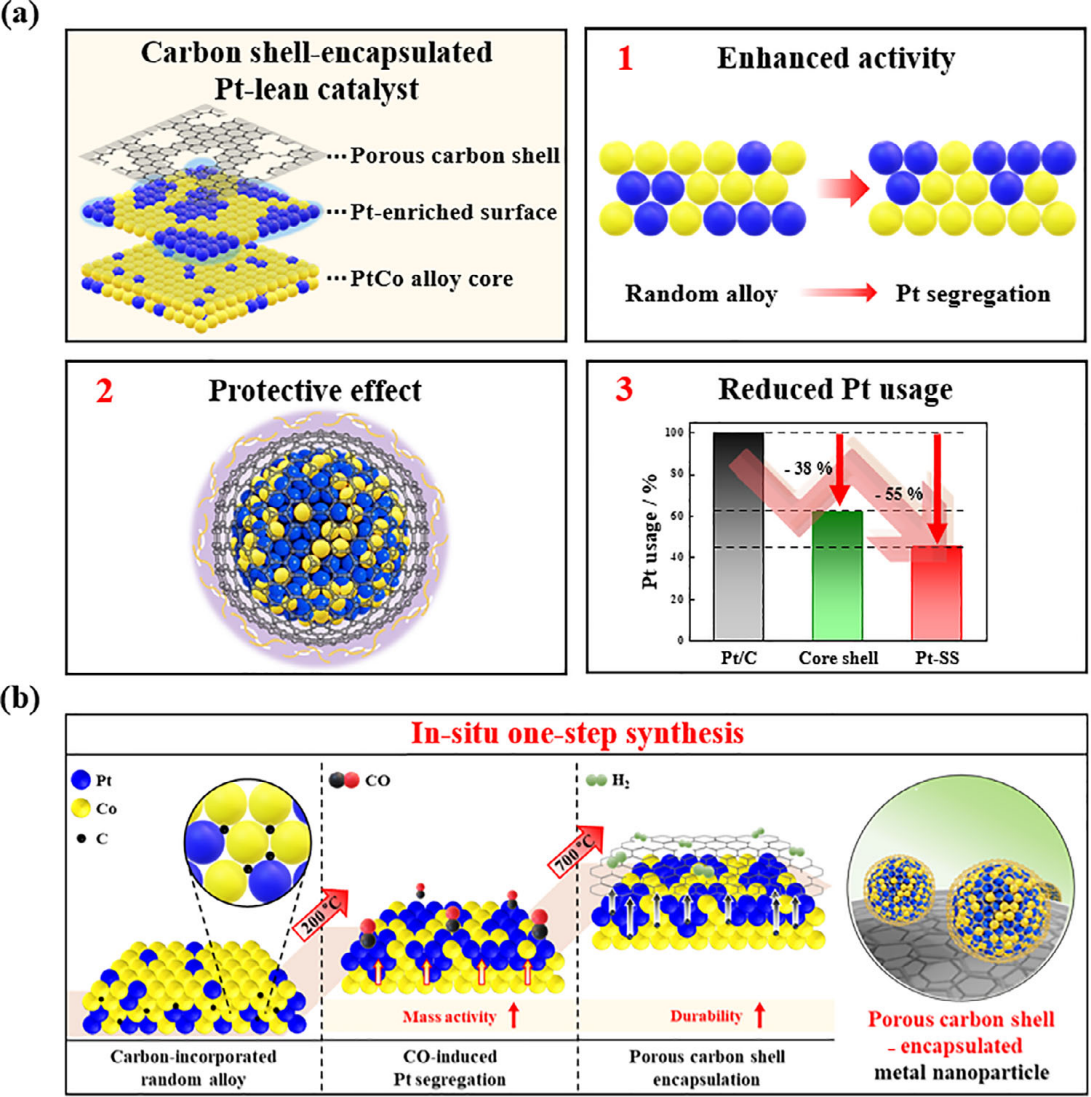
Schematics of (a) structure design and key advantages of the carbon shell‐encapsulated Pt‐lean catalyst and (b) in situ one‐step synthesis for Pt segregation and carbon shell encapsulation.

In this study, to significantly reduce Pt usage, we induced effective Pt segregation in low‐Pt alloy compositions to maximize catalytic activity, while simultaneously applying carbon shell encapsulation to enhance durability (Figure 1). The synthesized catalyst features a porous carbon shell‐encapsulated structure with a Pt‐segregated surface, exhibiting high ORR activity. The external carbon shell effectively prevents metal dissolution under electrochemical operating conditions. In contrast to conventional core–shell structures, this catalyst does not require a fully Pt‐covered core, enabling a substantial reduction in Pt loading to approximately 55% of that used in commercial catalysts. This is the result of adopting a distinctive in situ one‐step synthesis process, in contrast to conventional carbon shell coating strategies that rely on polymer coating followed by carbonization. This process enables carbon atoms to be absorbed into the metal lattice during synthesis, followed by heat treatment that simultaneously induces Pt segregation and the formation of a porous carbon shell encapsulation. This simple method streamlines the synthesis process by enabling simultaneous Pt segregation and carbon shell formation during catalyst fabrication. As a result, both cost reduction and simultaneous improvements in activity and durability were achieved. This strategy demonstrates excellent performance even with reduced Pt usage, making it a promising approach for lowering the cost of fuel cell catalyst layers and enhancing their market competitiveness.

## 2 Results and Discussion

### 2.1 In situ One‐Step Process to Simultaneously Control Surface Alloy and Carbon Shell Structures

**FIGURE 2:**
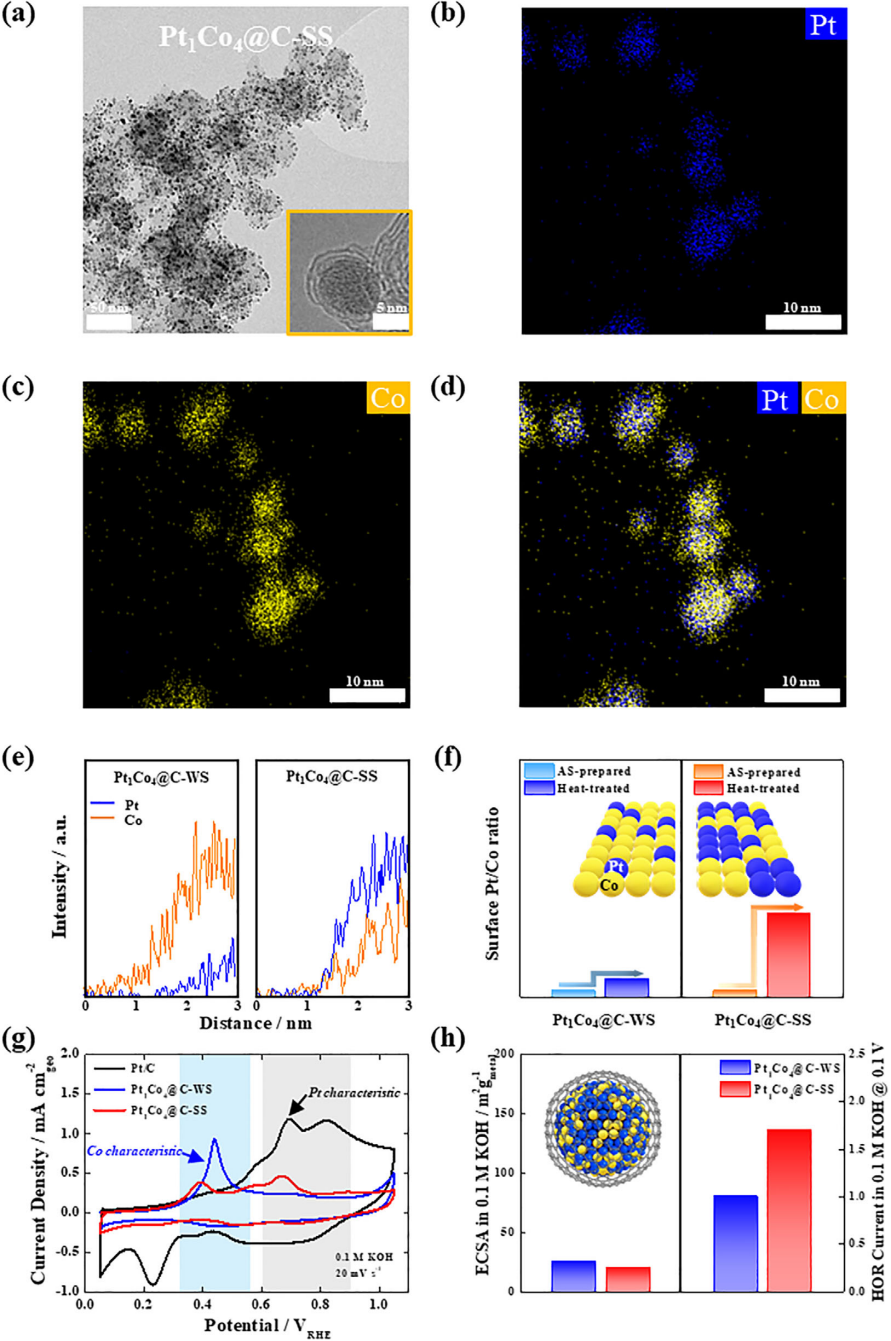
(a) TEM image of Pt_1_Co_4_@C/C‐SS catalyst. Inset: HR‐TEM image of carbon shell formed on the surface of the nanoparticle. EDS mapping images of (b) Pt (blue dots), (c) Co (green dots), and (d) both Pt and Co atoms. (e) Line profile data and (f) Pt and Co atomic ratio in XPS analysis of Pt_1_Co_4_@C/C‐WS and Pt_1_Co_4_@C/C‐SS. (g) CO stripping curves in KOH of Pt/C, Pt_1_Co_4_@C/C‐WS, and Pt_1_Co_4_@C/C‐SS. (h) Correlation between the ECSA and HOR current in KOH of Pt_1_Co_4_@C/C‐WS and Pt_1_Co_4_@C/C‐SS.

The carbon shell‐encapsulated PtCo alloy nanoparticle catalyst was fabricated through a solvothermal reaction, followed by an in situ one‐step annealing process utilizing CO and H_2_ gases. During the solvothermal synthesis, the thermal decomposition of Pt(acac)_2_ and Co(acac)_2_ precursors generates a small amount of carbon source, which is subsequently absorbed into the metal nanoparticle lattice. First of all, during the in situ one‐step annealing process using CO and H_2_ gases (Figure 1a), carbon atoms absorbed into the metal lattice form a carbon shell on the surface of the metal nanoparticles through segregation and carbonization at 700 °C (Figure S2), which is clearly confirmed in the inset of Figure 2a [24, 25, 26, 27, 28, 29, 30, 31, 32, 33]. At the same time, CO annealing at 200 °C in the continuous process enables elaborate modulation of the surface alloy structure. The strong binding energy of CO with the Pt atom promotes surface segregation of Pt within the PtCo alloy structure at 200 °C [34, 35, 36, 37, 38], whereas H_2_ gas induces etching of the carbon shell at 700 °C [39, 40], facilitating the formation of a porous carbon shell (Figure 1b). Therefore, it is concluded that the in situ one‐step annealing process using CO and H_2_ gases simultaneously controls the alloy structure and the carbon shell structure on the nanoparticle surface within a single thermal treatment step, leveraging the distinct roles of each gas.

### 2.2 Structure Characterization of PtCo@C/C Catalysts

The structural characteristics of the synthesized Pt_1_Co_4_@C/C‐SS catalyst were analyzed to confirm the effects of the proposed synthesis and heat treatment processes. TEM image and energy‐dispersive X‐ray spectroscopy (EDS) mapping analyses revealed that the catalyst exists as uniformly dispersed nanoparticles on the carbon support. Additionally, the carbon shell was observed on the nanoparticle surface, with Co concentrated in the core and Pt preferentially segregated at the surface (Figure 2a–d; Figure S2). Consistent with these observations, X‐ray diffraction (XRD) analysis further confirmed the formation of a Pt–Co alloy phase, as shown in Figure S3.

Furthermore, to verify the effect of the in situ one‐step process using CO and H_2_ gases in the fabrication of the Pt_1_Co_4_@C/C‐SS catalyst, a conventional heat treatment process was conducted using the Pt_1_Co_4_@C‐ASP (As‐Prepared) catalyst. For this purpose, heat treatment was conducted using only H_2_ gas without CO annealing at 200 °C, resulting in the formation of the Pt_1_Co_4_@C‐WS (Weak Segregation) catalyst (Figure S4). EDS line scanning analysis demonstrated distinct elemental distributions between the two catalysts. In Pt_1_Co_4_@C‐WS, Co remained concentrated at the surface, indicating a lack of Pt segregation, whereas in Pt_1_Co_4_@C/C‐SS, Pt was predominantly enriched at the surface due to the segregated effect of the in situ one‐step process (Figure 2e). These tendencies were further corroborated by XPS analysis, where a significant increase in the surface Pt/Co ratio was observed in Pt_1_Co_4_@C/C‐SS compared to the Pt_1_Co_4_@C‐ASP sample, as is without any heat treatment, while no substantial change was detected in Pt_1_Co_4_@C‐WS (Figure 2f; Figure S5). This confirms that the in situ one‐step annealing process effectively promotes Pt surface segregation.

Electrochemical analysis results further support these structural characteristics. CO stripping experiments conducted under an alkaline condition revealed distinct electrochemical behaviors: Pt_1_Co_4_@C‐WS exhibited characteristic peaks associated with the Co redox reactions due to its surface enrichment, whereas Pt_1_Co_4_@C/C‐SS indicated a more Pt‐like electrochemical characteristic similar to that of commercial Pt/C catalysts (Figure 2g). Although the ECSA values of Pt_1_Co_4_@C‐WS and Pt_1_Co_4_@C/C‐SS were comparable, hydrogen oxidation reaction (HOR) activity in alkaline conditions was significantly higher for Pt_1_Co_4_@C/C‐SS, further confirming the effect of Pt segregation (Figure 2h; Figure S6). Collectively, TEM, XPS, and electrochemical analyses provide clear evidence that the in situ one‐step annealing process plays a critical role in enhancing Pt surface segregation in Pt_1_Co_4_@C/C‐SS.

### 2.3 Activity and Durability Tests for PtCo@C/C Catalysts in Half‐Cells

**FIGURE 3:**
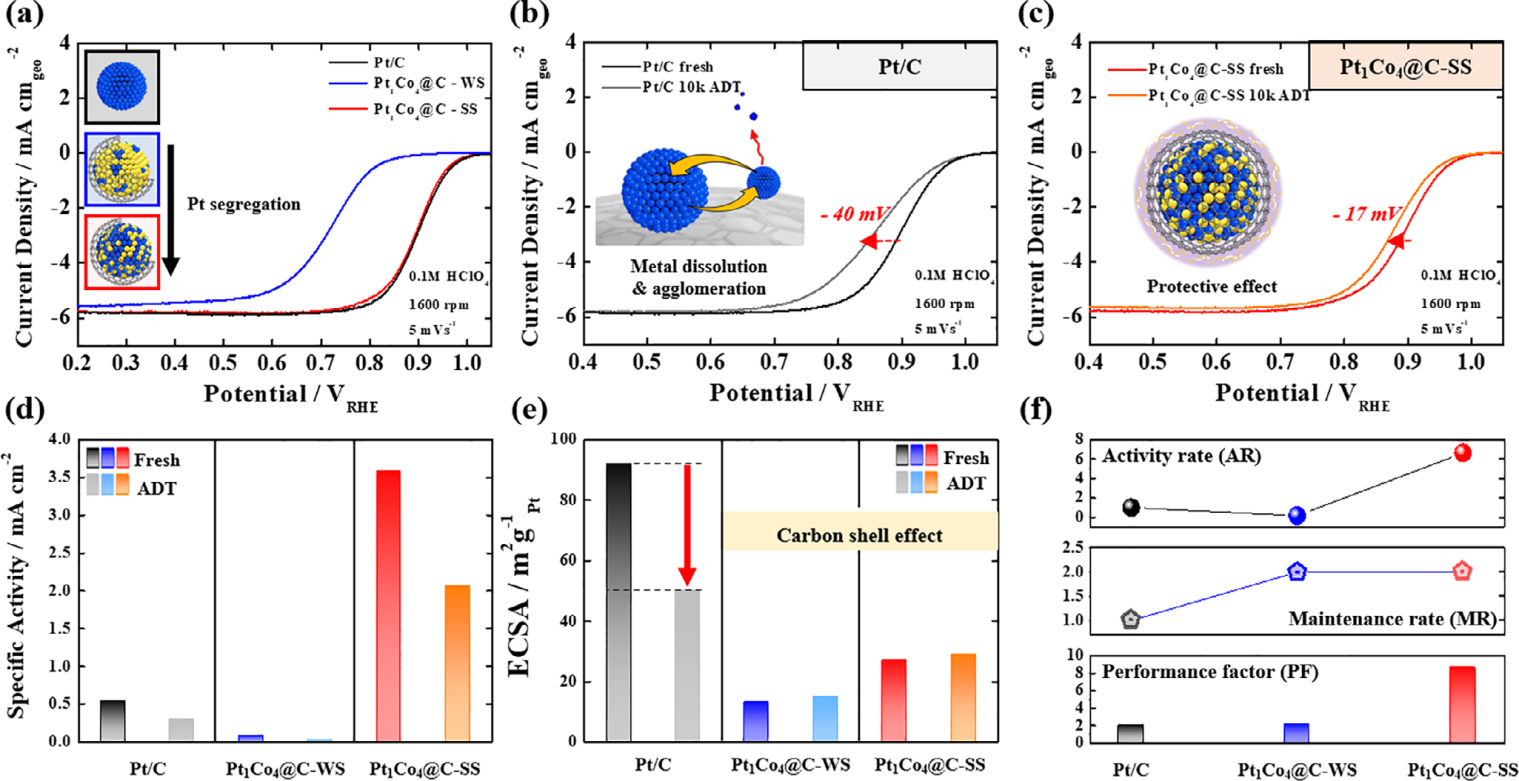
Half‐cell data of Pt/C, Pt_1_Co_4_@C/C‐WS, and Pt_1_Co_4_@C/C‐SS. (a) ORR polarization curves of catalysts. Accelerated stress test (AST) results of (b) Pt/C and (c) Pt_1_Co_4_@C/C‐SS. (d) Specific activity, (e) ECSA, and (f) activity rate (AR), maintenance rate (MR), and performance factor (PF) of Pt/C, Pt_1_Co_4_@C/C‐WS, and Pt_1_Co_4_@C/C‐SS. The AR represents the specific activity (SA) of each catalyst normalized to that of the commercial Pt/C catalyst. The MR indicates the change in ECSA before and after the ADT, also normalized to the commercial Pt/C catalyst. Finally, the PF, defined as the sum of AR and MR, serves as an index reflecting the overall performance of the catalyst.

The synergistic effect of the Pt segregation structure and the porous carbon shell was further validated through ORR activity measurements conducted under acidic conditions relevant to polymer electrolyte membrane fuel cells (PEMFCs). ORR polarization curves revealed a distinct contrast between catalysts subjected to conventional and in situ one‐step fabrication processes. Pt_1_Co_4_@C‐WS exhibited significantly lower ORR activity, whereas Pt_1_Co_4_@C/C‐SS demonstrated high ORR activity comparable to that of commercial Pt/C catalysts (Figure 3a). This result can be attributed to the efficient utilization of Pt as active sites in the Pt_1_Co_4_@C/C‐SS catalyst through Pt segregation, despite the lower Pt content. In contrast, the Pt_1_Co_4_@C‐WS catalyst, with a random alloy structure, retains a significant portion of Pt inside the nanoparticles, preventing it from serving as active sites (Figure 1a; Table S1).

In addition to its high activity, Pt_1_Co_4_@C/C‐SS exhibited remarkable durability, benefiting from the protective effect of the carbon shell. While commercial Pt/C catalysts initially showed high ORR activity, severe activity degradation was observed after a 10,000‐cycle durability test. Specifically, the half‐wave potential (E_1/2_) of Pt/C decreased by 40 mV, and TEM analysis revealed extensive nanoparticle aggregation, indicative of structural degradation (Figure 3b; Figure S7). In contrast, Pt_1_Co_4_@C/C‐SS exhibited significantly improved stability, with only a 17 mV decrease in E_1/2_, corresponding to less than half of the degradation observed in Pt/C catalysts (Figure 3c; Figure S7). Furthermore, TEM images confirmed that the carbon shell remained, preserving nanoparticle dispersion even after the durability test. EDS mapping analysis before and after durability test showed that the Pt/Co atomic ratio remained nearly unchanged, supporting the conclusion that the carbon shell plays a crucial role in mitigating catalyst degradation (Figure S8).

The advantages of the Pt segregation structure and carbon shell were further demonstrated by specific activity (SA), mass activity (MA), and ECSA comparisons. While both Pt_1_Co_4_@C‐WS and Pt_1_Co_4_@C/C‐SS catalysts exhibited relatively low ECSA values due to carbon shell coverage, Pt_1_Co_4_@C/C‐SS indicated a significantly higher SA and MA than commercial Pt/C catalysts, owing to the synergistic effects of Pt segregation and the porous carbon shell (Figure 3d; Figure S9). Additionally, despite its lower ECSA, Pt_1_Co_4_@C/C‐SS retained a stable ECSA after durability test, highlighting the protective role of the carbon shell (Figure 3e; Figure S10).

A comprehensive performance measurement further established the superior catalytic properties of Pt_1_Co_4_@C/C‐SS relative to commercial Pt/C. The catalyst exhibited an activity rate (AR) 6.6 times higher than Pt/C and a maintenance rate (MR) twice as high, leading to an overall performance factor (PF) that was 4.3 times greater (Figure 3f). These results demonstrate that the synergistic effect of Pt segregation and the porous carbon shell, induced by in situ one‐step annealing process, plays a pivotal role in maximizing both catalytic activity and durability while significantly reducing Pt usage [27, 29, 41].

### 2.4 MEA Performance and Accelerated Stress Test (AST)

**FIGURE 4:**
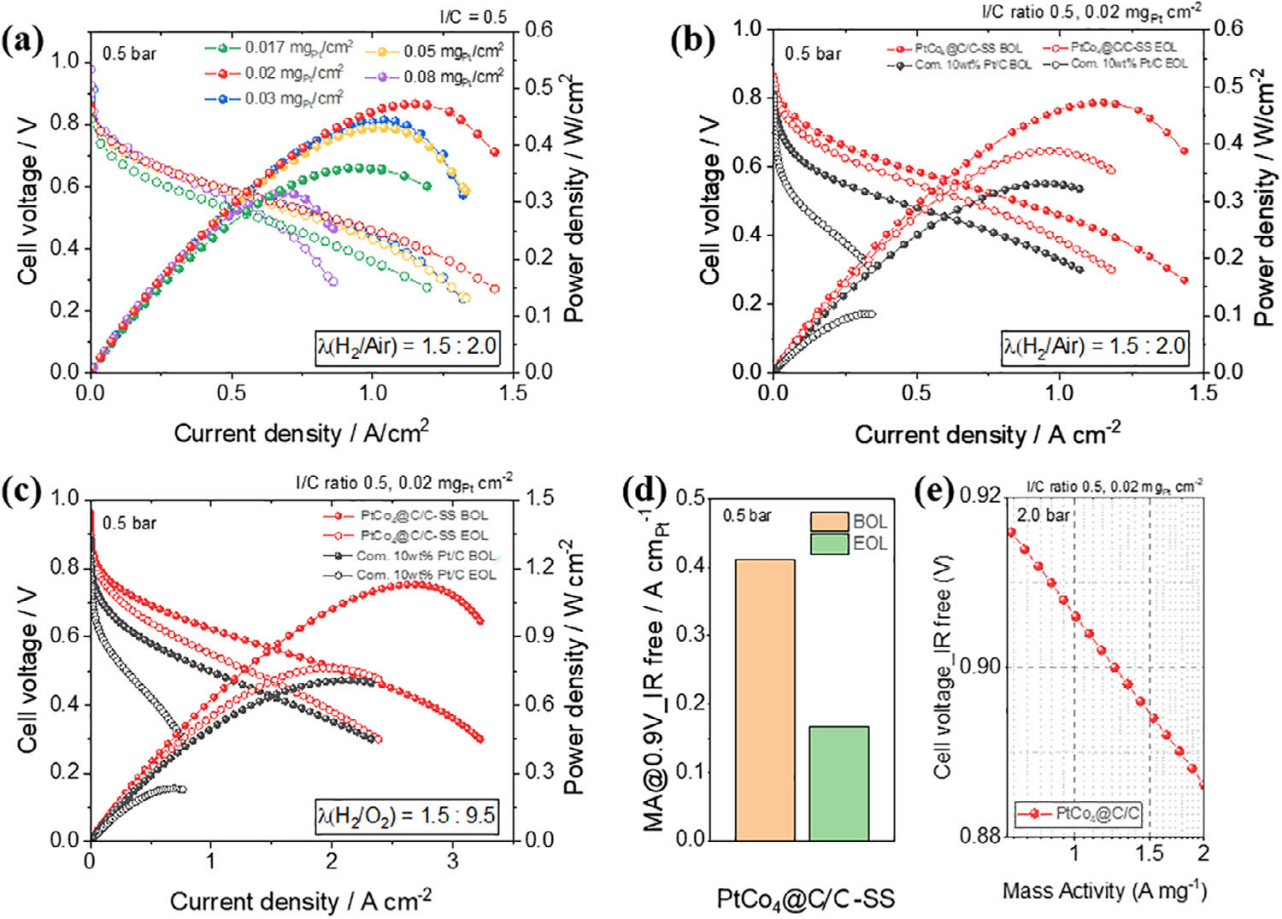
Performance and durability of ultralow Pt loading MEAs using Pt_1_Co_4_@C/C‐SS and commercial Pt/C in PEMFC single cells (a) Polarization curves with varying Pt loadings under H_2_/air. Before and after the DOE AST protocol, polarization curves of Pt_1_Co_4_@C/C‐SS and commercial Pt/C under (b) H_2_/air (λ = 1.5/2.0), (c) H_2_/O_2_ (λ = 1.5/9.5). (d) MA at 0.9 V (IR‐free) for Pt_1_Co_4_@C/C‐SS before and after AST. (e) Correlation between IR‐free cell voltage and MA at 0.9 V under H_2_/air (2.0 bar).

To validate the practical applicability of the synthesized Pt_1_Co_4_@C/C‐SS catalyst in PEMFCs, ultralow Pt loading MEAs were fabricated and evaluated against commercial Pt/C (10 wt.%) catalysts. Performance optimization was achieved by systematically varying the Pt loading density (0.017–0.08 mg_Pt_ cm^−2^) and ionomer‐to‐carbon (I/C) ratio (0.45–0.55), as shown in Figure 4a and Figure S11, under fully humidified H_2_/air conditions at 80 °C and 0.5 bar backpressure. Optimization of Pt loading density allowed balancing active site availability, catalyst layer thickness, and mass transport resistance, while tuning the I/C ratio adjusted the trade‐off between proton conductivity and gas diffusion [42]. Among the tested conditions, the MEA with 0.02 mg_Pt_ cm^−2^ and an I/C ratio of 0.5 achieved the best compromise between activity and Pt utilization and was selected for durability evaluation.

Figure 4b,c, and Figure S12 show MEA performance and durability before and after the 30,000‐cycle U.S. DOE AST protocol under H_2_/air and H_2_/O_2_, respectively. H_2_/air conditions represent realistic operation with mass transport limitations, while H_2_/O_2_ conditions minimize these limitations, enabling the accurate evaluation of intrinsic activity, including reliable MA measurements at high potential, 0.9 V. Thus, the combined use of these gas environments provides a comprehensive assessment of catalyst performance. Under both conditions, the Pt_1_Co_4_@C/C‐SS MEA demonstrated superior beginning‐of‐life (BOL) voltage and power density compared to commercial Pt/C. Remarkably, it retained a significant portion of its initial performance after AST, whereas commercial Pt/C showed greater degradation. These results highlight the excellent activity and durability of Pt_1_Co_4_@C/C‐SS under practical PEMFC conditions, consistent with trends observed in half‐cell measurements and supporting its real‐world applicability.

As shown in Figure 4d,e, and Figure S13, the Pt_1_Co_4_@C/C‐SS MEA achieved a MA exceeding 0.4 A mg_Pt_^−1^ at 0.9 V (IR‐free) under 0.5 bar backpressure, which decreased to ∼0.23 A mg_Pt_^−1^ after AST, corresponding to a 42.5% retention—surpassing the DOE 2025 target of 40%. At 2.0 bar backpressure, the MA further increased to 1.26 A mg_Pt_^−1^, indicating enhanced oxygen transport and catalyst utilization under pressurized conditions. In contrast, commercial Pt/C exhibited both lower initial MA and more severe degradation. Due to unmeasured current at 0.9 V under 0.5 bar, MA for commercial Pt/C was instead evaluated at 0.85 V for BOL and end of life (EOL), where Pt_1_Co_4_@C/C‐SS still outperformed in both activity and retention (Figure S13). The correlation between MA and IR‐free voltage further underscores the superior ORR kinetics of the developed catalyst across diverse operating conditions (Figure 4e).

Post‐AST structural characterization using TEM, HAADF‐STEM, and EDS mapping (Figure S14) revealed that PtCo nanoparticles in the Pt_1_Co_4_@C/C‐SS catalyst remained well‐confined within carbon shells even after 30,000 durability cycles. Notably, TEM‐EDS analysis showed that the Co content decreased to approximately 40% of its initial value, suggesting substantial Co leaching during the AST. While such analysis is inherently localized and may not fully reflect the bulk composition, it clearly indicates the occurrence of Co dissolution, which is likely one of the contributors to the observed performance degradation in MEAs. Despite this, the extent of nanoparticle growth, aggregation, and metal dissolution in PtCo nanoparticles was significantly less severe than that observed in the commercial Pt/C catalyst, as shown in Figures S14 and S15. In particular, commercial Pt/C exhibited considerable structural deterioration, including complete Pt dissolution in some regions and substantial particle agglomeration, underscoring the protective function of the carbon shell. These comparative results suggest that the carbon shell in Pt_1_Co_4_@C/C‐SS does not completely block degradation pathways but plays a critical role in mitigating them. By physically confining the nanoparticles and providing a chemical barrier, the carbon shell effectively suppresses excessive metal leaching and sintering, thereby preserving the catalyst's structural integrity and electrochemical performance under harsh PEMFC operating conditions.

By effectively anchoring the active sites and mitigating both metal dissolution and nanoparticle agglomeration, the carbon shell enabled excellent initial MA and practically relevant MA durability, even at an ultralow Pt loading. This structural feature helped address the inherent durability limitations of the Co‐rich core material, allowing the catalyst to meet the DOE 2025 target of 40% MA retention. Consequently, despite its lower Pt content, the Pt_1_Co_4_@C/C‐SS catalyst exhibited electrochemical performance and durability that rivaled commercial Pt/C, underscoring the effectiveness of the carbon shell‐based architecture.

These results highlight the dual function of the carbon shell: (1) enhancing ORR activity by improving electrical conductivity and ensuring uniform dispersion of PtCo nanoparticles, and (2) mitigating structural degradation by physically confining the active nanoparticles and buffering against the acidic PEMFC environment. While the carbon shell does not completely eliminate metal dissolution or nanoparticle growth during AST, it effectively suppresses Co leaching and inhibits particle sintering and agglomeration—two primary degradation mechanisms in low‐Pt, Co‐rich PtCo alloy systems [43, 44, 45]. This stabilization accounts for the high MA retention observed after durability testing under severe operating conditions. Consequently, despite the relatively low Pt content, the Pt_1_Co_4_@C/C‐SS catalyst exhibits superior MA and durability due to the synergistic effect of Pt surface segregation and porous carbon shell encapsulation, which collectively stabilize the Co‐rich core and protect the catalytic interface under harsh PEMFC conditions.

## 3 Conclusions

In this study, we developed a novel ultralow‐Pt‐loading catalyst, Pt_1_Co_4_@C/C‐SS, composed of Co‐rich PtCo alloy nanoparticles encapsulated within a porous carbon shell. Through a facile in situ one‐step annealing process using CO and H_2_ gases, we successfully achieved both Pt surface segregation and carbon shell formation in a single heat treatment. Structural characterizations, including TEM, EDS, and XPS, confirmed the formation of a Pt‐enriched surface and uniform carbon shell encapsulation, which synergistically contributed to both activity and durability. Electrochemical analysis revealed that the Pt_1_Co_4_@C/C‐SS catalyst exhibited excellent ORR activity, comparable to that of a commercial Pt/C, despite its significantly reduced Pt content (up to 55% reduction in Pt content). The catalyst also demonstrated superior durability under AST conditions than commercial Pt/C catalyst. Notably, even with a much lower Pt content, the Pt_1_Co_4_@C/C‐SS catalyst maintained structural and electrochemical stability, outperforming Pt/C in both half‐cell and MEA configurations. The MEA fabricated with Pt_1_Co_4_@C/C‐SS achieved a BOL MA exceeding 0.4 A mg_Pt_^−1^ under 0.5 bar conditions, and up to 1.26 A mg_Pt_^−1^ under 2.0 bar, retaining 42.5% of its activity after 30,000 AST cycles—surpassing the U.S. DOE 2025 durability target (40%). These performance metrics, alongside the significantly reduced Pt usage (55% of commercial levels), highlight the effectiveness of the carbon shell design in balancing high ORR activity and catalyst durability. Therefore, we believe that the Pt_1_Co_4_@C/C‐SS catalyst presents a promising strategy for next‐generation PEMFC cathodes, addressing the dual challenges of cost reduction and long‐term durability. This work offers meaningful insights into the structural design of alloy/carbon hybrid catalysts and opens new avenues for the fabrication of high‐performance electrocatalysts.

## 4 Experimental Section

### 4.1 Chemicals and Materials

Carbon blacks (Vulcan XC72, Cabot) were purchased from Cabot Inc. (Alpharetta, GA, USA). 1‐Octadecene (90%), platinum acetylacetonate (Pt(acac)2, 97%), cobalt acetylacetonate (Co(acac)2, 97%), oleylamine (70%), Nafion ionomer (5 wt.%), and 2‐propanol (99.5%) were procured from Sigma–Aldrich (St. Louis, MO, USA). n‐Hexane (95%) and ethanol (95%) were acquired from Samchun Pure Chemicals (Daejeon, Korea). A rotating disk electrode (RDE) with glassy carbon (0.196 cm^2^) was purchased from Metrohm‐Autolab (Netherlands).

### 4.2 Catalyst Preparation

In a vial, 0.0235 g of Pt (acac)_2_, 0.0615 g of Co (acac)_2_, 5 mL of oleylamine, and 30 mL of 1‐octadecene were mixed via ultrasonication for 20 min. Using a three‐necked flask, 0.1 g of carbon black and 10 mL of oleylamine were also dispersed in 130 mL of 1‐octadecene through ultrasonication for 20 min. After the metal precursor solution in the vial was transferred to a three‐necked flask, it was sonicated for 5 min. The solution temperature was then raised to 300 °C in the flask and maintained for 2 h for pyrolysis of the metal precursors. After the solvothermal reaction was complete, the solution was cooled to 80 °C and filtered using n‐hexane and ethanol for washing. The as‐prepared catalysts were dried in a vacuum oven at 60 °C. To fabricate the carbon shell‐coated Pt‐segregated catalyst, a one‐step heat treatment process was conducted at 200 °C in a CO gas atmosphere, followed by heat treatment at 700 °C in 10% H_2_/N_2_ mixed gas atmosphere. The total metal loading of the synthesized catalyst was approximately 20 wt.%, and the Pt loading was approximately 9 wt.% (Figure S16).

### 4.3 Physical Characterization

The dispersion and average particle size of catalysts measured by transmission electron microscope (TEM) (Tecnai G2 F30S‐ Twin, FEI) were confirmed, and the carbon layer coated on the surface of nanoparticles was clearly observed using high‐resolution TEM (HR‐TEM) (JEOL 2010 FasTEM microscope). Furthermore, the electronic structures of catalysts were observed using X‐ray photoelectron spectroscopy (XPS) (K‐alpha+, Thermo Scientific Co.). Finally, the composition of the PtCo alloy was confirmed to be 1:4 through Inductively Coupled Plasma Atomic Emission Spectroscopy (ICP‐AES) (OPTIMA 7300 DV, Perkin‐Elmer) analysis. The metal loading of the catalysts was measured by a Thermogravimetric analyzer (TGA) (TGA N‐1000, SCINCO).

### 4.4 Electrochemical Measurements

All electrochemical measurements were performed in a standard three‐compartment electrochemical cell with a RDE, Pt wire, and Ag/AgCl electrode as the working, counter, and reference electrodes, respectively. Herein, all potential values were represented by a reversible hydrogen electrode (RHE). The catalyst inks were prepared by mixing 5 mg of the catalyst with 68.7 µL of Nafion solution and 500 µL of 2‐propanol. The catalyst loading on the glassy carbon of RDE was 44.86 µg_metal_ cm^−2^ for all catalysts. In Ar‐saturated 0.1 m KOH and 0.1 m HClO_4_, cyclic voltammograms (CVs) were scanned in the potential range of 0.05–1.05 V at 20 mV s^−1^. In addition, CO stripping tests were performed to determine the electrochemical surface area (ECSA) values of the catalysts by integrating the CO oxidation current. While the working electrode potential was kept at 0.05 V, 99.95% CO gas was first bubbled for 15 min in the electrolyte for CO adsorption on the Pt surface, and then was replaced by Ar to remove the CO residue from the electrolyte. After Ar bubbling for 15 min, CO stripping was conducted at a scan rate of 20 mV s^−1^ in the potential range of 0.05–1.05 V. The hydrogen oxidation reaction (HOR) performance was evaluated in H_2_‐saturated 0.1 m KOH at a scan rate of 5 mV s^−1^ and a rotation speed of 1600 rpm in the potential range of 0.0–1.05 V. For oxygen reduction reaction (ORR), the polarization curves were obtained in O_2_‐saturated 0.1 m HClO_4_ at a scan rate of 5 mV s^−1^ and a rotation speed of 1600 rpm in the potential range of 0.05–1.05 V. The accelerated degradation test (ADT) of the fabricated catalyst performed 10,000 potential cycle CV in the range of 0.6 V_RHE_ and 1.1 V_RHE_ at a scan rate of 100 mV s^−1^ while injecting oxygen gas into 0.1 m HClO_4_. After the accelerated deterioration evaluation was completed, CV, ORR, and CO stripping were measured for each sample, and durability was observed by comparing them with the data before the accelerated deterioration evaluation. In addition, for comparison with the fabricated catalyst, the same analysis as above was performed using commercial Pt/C (Premetek). Additionally, the activity rate (AR), maintenance rate (MR), and performance factor (PF) were calculated based on electrochemical tests to enable a comprehensive comparison of the catalytic performance. The AR represents the specific activity (SA) of each catalyst normalized to that of the commercial Pt/C catalyst. The MR indicates the change in ECSA before and after the ADT, also normalized to the commercial Pt/C catalyst. Finally, the PF, defined as the sum of AR and MR, serves as an index reflecting the overall performance of the catalyst.

### 4.5 Membrane Electrode Assembly (MEA) Fabrication

All MEAs were fabricated via the catalyst‐coated membrane (CCM) method using an automated spray system integrated with a Sono‐Tek ultrasonic nozzle, following our previously reported procedure [46]. Catalyst inks were prepared by dispersing the synthesized catalyst or commercial Pt/C (10 wt.%, Johnson Matthey), along with Vulcan carbon, in a mixture of Aquivion D72‐25BS ionomer (Sigma–Aldrich) and isopropyl alcohol, followed by ultrasonication to achieve a homogeneous solution. The resulting inks were directly sprayed onto a Nafion 211 membrane (DuPont, thickness = 25.4 µm), a commercial perfluorosulfonic acid‐based proton exchange membrane. Pt_1_Co_4_@C/C‐SS was employed as the cathode catalyst, whereas commercial Pt/C was used for both the anode and the control MEA cathode. The cathode Pt loading was varied in the range of 0.017–0.08 mg_Pt_ cm^−^^2^ to optimize performance, while the anode Pt loading was fixed at 0.05 mg_Pt_ cm^−^^2^ for all samples. The ionomer‐to‐carbon (I/C) mass ratio was adjusted between 0.45 and 0.55 for the cathode, with the Pt loading fixed at 0.05 mg_Pt_ cm^−^^2^, while the I/C ratio for the anode was maintained at 0.6 for all MEAs. Each MEA had an active geometric area of 5 cm^2^. The prepared MEA was laminated with gas diffusion layers (GDL, JNTG‐30‐A6H), gaskets, bipolar plates, and end plates to assemble a single‐cell device. GDLs were cut to match the MEA active area, while gaskets were shaped to the membrane and bipolar plate dimensions, leaving only the active area exposed at the center.

### 4.6 Fuel Cell Testing

Fuel cell performance and catalyst durability were evaluated using a test station (CNL) under conditions based on U.S. Department of Energy (DOE) protocols [47]. Fully humidified H_2_/O_2_ or H_2_/air gases were supplied at 80 °C, and the cell temperature was also maintained at 80 °C. Backpressures of 0.5 bar and 2.0 bar were applied during testing. Prior to polarization measurements, MEAs were activated under H_2_/air conditions by applying cyclic voltammetry (CV) between open circuit voltage (OCV) and 0.3 V overnight. Current–voltage (I–V) polarization curves were recorded under controlled gas flow conditions, with a hydrogen‐to‐oxygen stoichiometry of 1.5:2 for H_2_/air operation and 2:9.5 for H_2_/O_2_ operation to ensure oxygen‐rich conditions. The platinum mass activity (MA) was calculated from the current density at 0.9 V under H_2_/O_2_ conditions, with voltage correction applied for both IR‐drop and H_2_ crossover. MEA durability was evaluated following the U.S. DOE accelerated stress test (AST) protocol for catalyst stability, in addition to performance measurements. Specifically, the MEA was subjected to 30,000 square‐wave potential cycles between 0.6 V_RHE_ and 0.9 V_RHE_, with a 3‐second hold at each potential, under H_2_/N_2_ conditions.

## Author Contributions

J.M. and K.K. carried out catalyst synthesis, half‐cell electrochemical measurements, physical characterization, and manuscript preparation. J.H.L. performed single‐cell MEA fabrication and testing, physical characterization, and contributed to manuscript writing. Y.K. and H.P. assisted with synthesis, data collection, and analysis. M.K. and D.L. supported additional characterization and validation. S.‐D.Y. and Y.S.K. provided conceptual guidance, contributed to data interpretation, and critically revised the manuscript. J.T.H., S.J.Y., and N.J. conceived and supervised the project, secured funding, and contributed to manuscript writing and editing. All authors discussed the results and approved the final manuscript.

## Conflicts of Interest

The authors declare no conflicts of interest.

## Supporting information




**Supporting File**: smll72693‐sup‐0001‐SuppMat.docx.

## Data Availability

The data that support the findings of this study are available from the corresponding author upon reasonable request.
