# Supplementary material for: In Situ Surface Reconstruction and Carbon Encapsulation for High‐Performance Pt‐Lean Catalysts beyond Conventional Core–Shell Designs
Source: Small. 2026 Feb 11;22(24):e11516. doi: 10.1002/smll.202511516 (PMC13114495; doi:10.1002/smll.202511516)
Supplement: Supplementary file 1 — Supporting File: smll72693‐sup‐0001‐SuppMat.docx. [file SMLL-22-e11516-s001.docx]

Supporting Information

**In-Situ Surface Reconstruction and Carbon Encapsulation for High-Performance Pt-Lean Catalysts beyond Conventional Core–Shell Designs**

*Jiho Min,^‡^ Jeong Hee Lee,^‡^ Keonwoo Ko,^‡^ Yunjin Kim, Hyelim Park, Mansu Kim, Dongwook Lee, Sung-Dae Yim, Yun Sik Kang,* Joseph T. Hupp,* Sung Jong Yoo,* and Namgee Jung**

J. Min, S.-D. Yim, Y. S. Kang

Hydrogen Fuel Cell Laboratory, Korea Institute of Energy Research (KIER), Daejeon 34129, Republic of Korea.

E-mail: [arided1@kier.re.kr](mailto:arided1@kier.re.kr)

J. H. Lee, D. Lee, S. J. Yoo

Center for Hydrogen‧Fuel Cell Research, Korea Institute of Science and Technology (KIST), Seoul 02792, Republic of Korea.

E-mail: [ysj@kist.re.kr](mailto:ysj@kist.re.kr)

K. Ko, Y. Kim, H. Park, N. Jung

Graduate School of Energy Science and Technology (GEST), Chungnam National University (CNU), 99 Daehak-ro, Yuseong-gu, Daejeon, 34134, Republic of Korea.

E-mail: [njung@cnu.ac.kr](mailto:njung@cnu.ac.kr)

K. Ko, M. Kim, J. T. Hupp

Department of Chemistry, Northwestern University, Evanston, Illinois, 60208 USA.

E-mail: [j-hupp@northwestern.edu](mailto:j-hupp@northwestern.edu)

**Table of Contents**

**Table S1.** ……………………………………………………………………………………. 25

**Figures S1.** …………………………………………………………………………………...26

**Figures S2.** …………………………………………………………………………………...27

**Figures S3.** …………………………………………………………………………...............28

**Figures S4.** ……………………………………………………………………………….......29

**Figures S5.** …………………………………………………………………………………...30

**Figures S6.** …………………………………………………………………………………...31

**Figures S7.** ……………………………………………………………………….…………. 32

**Figures S8.** ………………………………………………………………………………….. 33

**Figures S9.** ……………………………………………………………………….…………. 34

**Figures S10.** ………………………………………………………………………………… 35

**Figures S11.** ………………………………………………………………………………… 36

**Figures S12.** ………………………………………………………………………………… 37 **Figures S13.** ………………………………………………………………………………… 38

**Figures S14.** ………………………………………………………………………………… 39

**Figures S15.** ………………………………………………………………………………… 40

**Figures S16.** ………………………………………………………………………………… 41

**Table S1.** The Pt loading on the GC electrode for the half cell test. (The Pt loading of Co@Pt core shell catalyst is simulated based on Figure S1.)

| **Catalyst** | **Pt usage** | **Pt usage ratio** |
| --- | --- | --- |
| **Pt/C** | **44.86 µg cm^-2^** | **100 %** |
| **Co@Pt (Core shell)** | **27.96 µg cm^-2^** | **62 %** |
| **Pt_1_Co_4_@C** | **20.31 µg cm^-2^** | **45 %** |


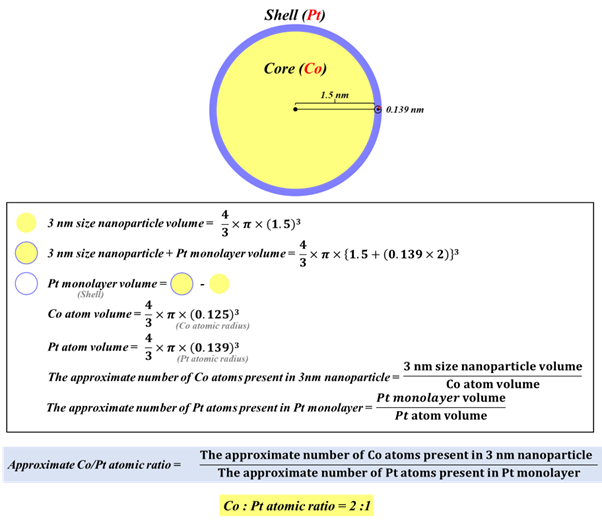


**Figure S1.** Calculation of the Co/Pt atomic ratio for Co@Pt core-shell catalyst.


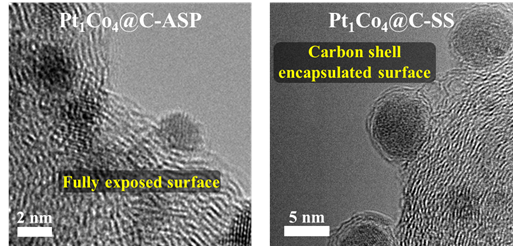


**Figure S2.** TEM images of Pt_1_Co_4_@C-ASP and Pt_1_Co_4_@C-SS (annealed).


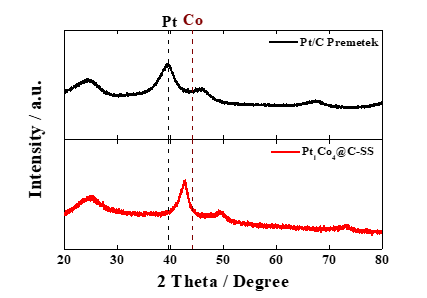


**Figure S3.** XRD patterns of Pt/C and Pt_1_Co_4_@C-SS.


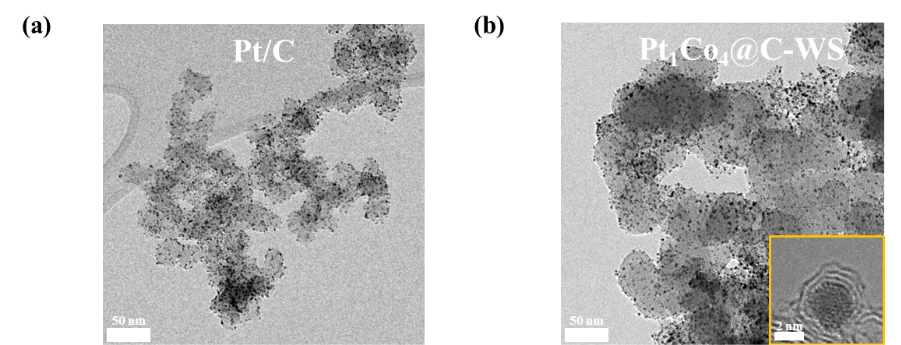


**Figure S4.** TEM images of (a) Pt/C and (b) Pt_1_Co_4_@C-WS. Inset: HR-TEM image of the carbon shell formed on the surface of nanoparticles.


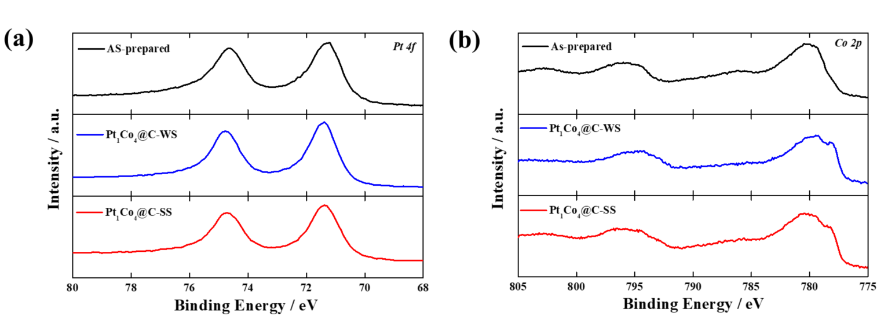


**Figure S5.** Comparison of (a) Pt 4f and (b) Co 2p XPS spectra of the prepared catalysts.


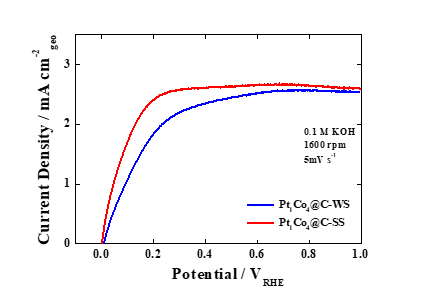


**Figure S6.** HOR polarization curves of Pt_1_Co_4_@C-WS and Pt_1_Co_4_@C-SS catalysts in KOH.


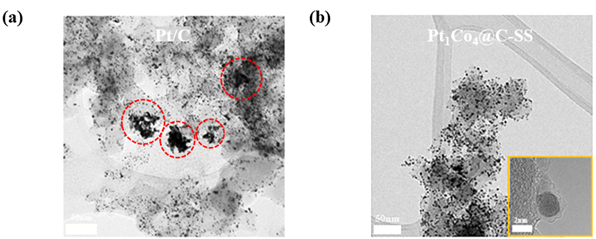


**Figure S7.** TEM images of (a) Pt/C and (b) Pt_1_Co_4_@C/C-SS after ADT in half-cell tests. Inset of (b): HR-TEM image of the carbon shell on the surface of a nanoparticle.


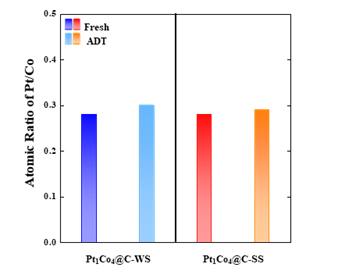


**Figure S8.** Pt/Co atomic ratio of Pt_1_Co_4_@C/C-WS and Pt_1_Co_4_@C/C-SS obtained from EDS mapping.


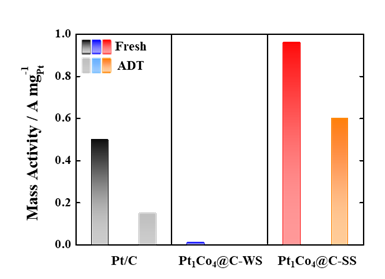


**Figure S9.** Mass activity ([@0.8](mailto:@0.8) V_RHE_) of Pt/C, Pt_1_Co_4_@C-WS, and Pt_1_Co_4_@C-SS.


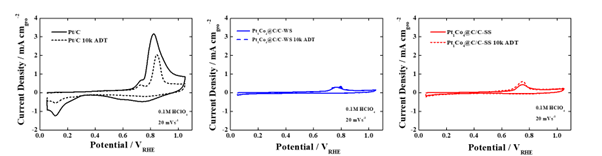


**Figure S10.** CO stripping curves of the prepared samples before and after ADT in 0.1 M HClO_4_.


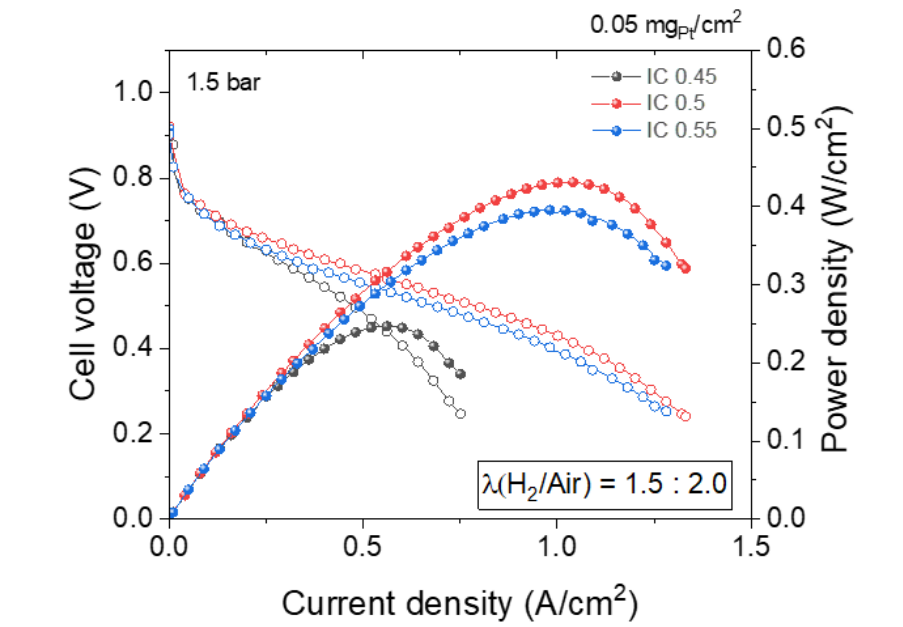


**Figure S11.** MEA performance test results conducted to optimize the ionomer-to-carbon (I/C) ratio for Pt_1_Co_4_@C/C-SS MEAs under H_2_/air conditions (λ = 1.5/2.0, 0.5 bar) at a fixed Pt loading of 0.05 mg_Pt_ cm⁻².


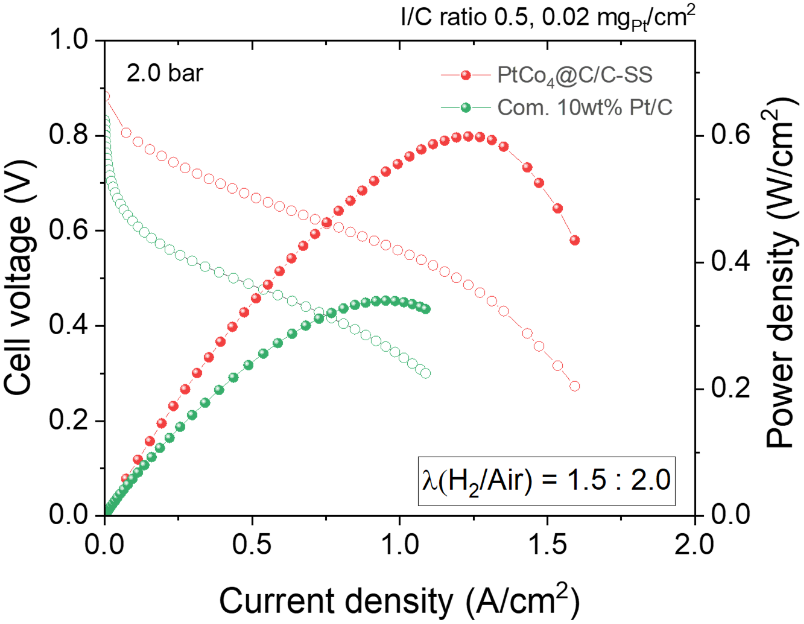


**Figure S12.** Polarization and power density curves of Pt_1_Co_4_@C/C-SS and commercial Pt/C MEAs under H_2_/air conditions (λ = 1.5/2.0) at 2.0 bar backpressure with Pt loading (0.02 mg_Pt_ cm^-2^) and I/C ratio (0.5).


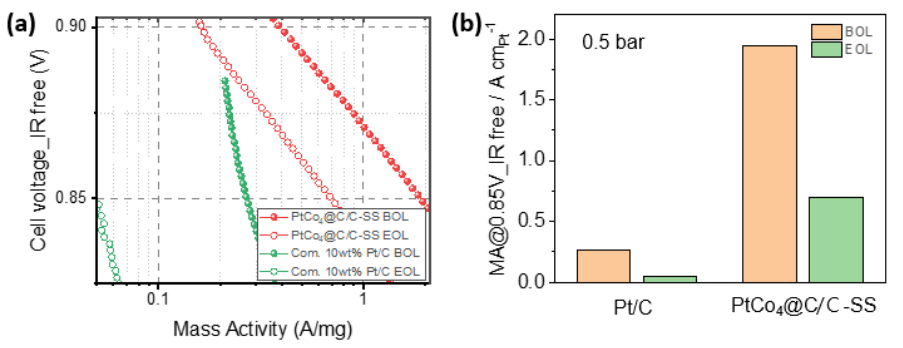


**Figure S13.** Comparison of IR-free mass activity between Pt_1_Co_4_@C/C-SS and commercial Pt/C at 0.5 bar. (a) Correlation between IR-free cell voltage and mass activity before and after AST. (b) Mass activity at 0.85 V (IR-free) for Pt/C and Pt_1_Co_4_@C/C-SS before (BOL) and after (EOL) AST.


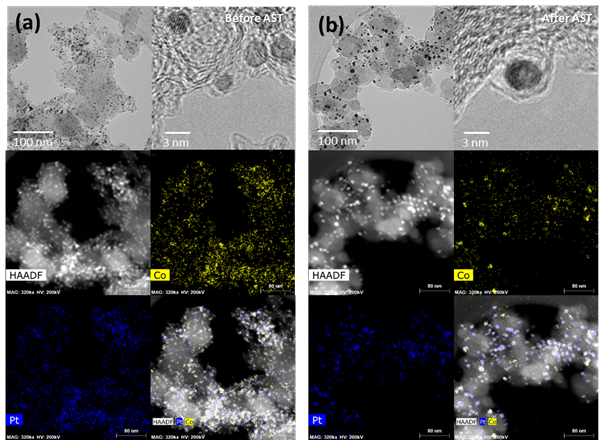


**Figure S14.** TEM, HAADF-STEM, and EDS mapping images of Pt_1_Co_4_@C-SS (a) before and (b) after 30,000-cycle MEA AST following the DOE protocol.


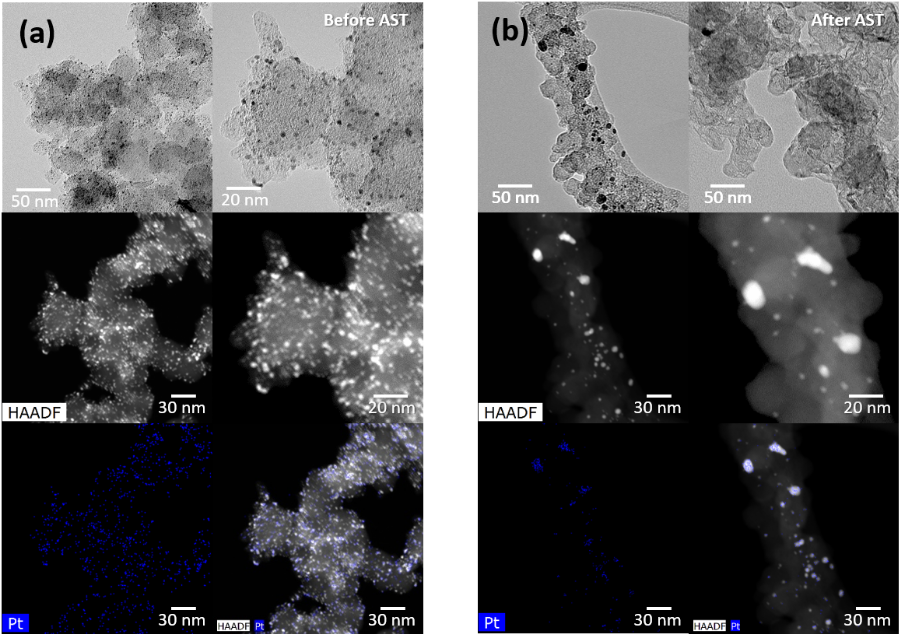


**Figure S15.** TEM, HAADF-STEM, and EDS mapping images of commercial Pt/C catalyst (10 wt% Pt) (a) before and (b) after 30,000-cycle MEA AST following the DOE protocol.


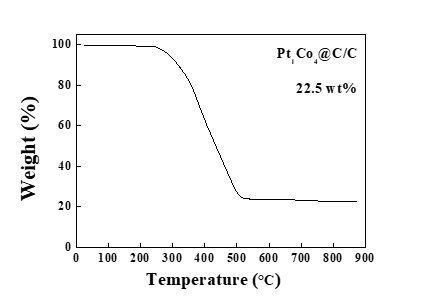


**Figure S16.** TGA graph of Pt_1_Co_4_@C.
